# Association of Microsatellite Instability and Gene Expression Profile in Colorectal Carcinoma and Potential Implications for Therapy

**DOI:** 10.3390/medicina60030348

**Published:** 2024-02-20

**Authors:** Muhammad G. Kibriya, Farzana Jasmine, Yuliia Khamkevych, Maruf Raza, Mohammed Kamal, Marc Bissonnette, Habibul Ahsan

**Affiliations:** 1 Institute for Population and Precision Health, Biological Sciences Division, The University of Chicago, Chicago, IL 60637, USA; farzana@uchicago.edu (F.J.); yuliiak@uchicago.edu (Y.K.); hahsan@bsd.uchicago.edu (H.A.); 2 Department of Public Health Sciences, Biological Sciences Division, The University of Chicago, Chicago, IL 60637, USA; 3 Department of Pathology, Jahurul Islam Medical College, Kishoregonj 2336, Bangladesh; drmarufraza@gmail.com; 4 Department of Pathology, The Laboratory, Dhaka 1205, Bangladesh; kamalzsr@yahoo.com; 5 Department of Medicine, The University of Chicago, Chicago, IL 60637, USA; mbissonn@bsd.uchicago.edu

**Keywords:** MSI, colorectal cancer, IL-17 signaling, immune check point inhibitors, gene expression, MMR deficiency

## Abstract

*Background and Objective:* In sporadic colorectal carcinomas (CRC), microsatellite instability (MSI) pathways play important roles. Previously, we showed differences in DNA methylation patterns in microsatellite stable (MSS) colorectal carcinomas and MSI-CRC. In the current study, we explore the similarities and differences in gene expression profiles in MSS and MSI at the gene level and at the pathway level to better understand CRC pathogenesis and/or the potential for therapeutic opportunities. *Material and Methods:* Seventy-one CRC patients (MSI = 18, MSS = 53) were studied. Paired tumor and adjacent normal tissues were used for genome-wide gene expression assays. *Result:* At the gene level, we compared the list of differentially expressed genes (fold change (FC) ≥ 3 and FDR < 0.05) in tumor tissues compared to corresponding normal tissue in CRC patients with MSI tumors (190 genes) and MSS tumors (129 genes). Of these, 107 genes overlapped. The list of genes that were differentially expressed in MSI tumors only showed enrichment predominantly in two broad categories of pathways—(a) Inflammation-related pathways including the interleukin-17 (*IL-17*) signaling pathway, tumor necrosis factor (*TNF*) signaling pathway, chemokine signaling, nuclear factor kappa B (*NFκB*) signaling, and cytokine-cytokine interactions, and (b) metabolism-related pathways, including retinol metabolism, steroid hormone biosynthesis, drug metabolism, pentose and glucoronate interconversions, and ascorbate and aldarate metabolism. The genes in inflammation-related pathways were up-regulated whereas genes in metabolism-related pathways were down-regulated in MSI tumor tissue. Pathway-level analysis also revealed similar results confirming the gene enrichment findings. For example, the 150 genes involved in the *IL-17* signaling pathway were on average up-regulated by 1.19 fold (CI 1.16–1.21) in MSI compared to 1.14 fold (CI 1.13–1.16) in MSS patients (interaction *p* = 0.0009). *Conclusions:* We document an association between MSI status and differential gene expression that broadens our understanding of CRC pathogenesis. Furthermore, targeting one or more of these dysregulated pathways could provide the basis for improved therapies for MSI and MSS CRC.

## 1. Introduction

In colorectal carcinomas (CRC), loss of genomic stability plays a key role that initiates tumorigenesis. This includes both chromosomal instability and microsatellite instability (MSI). This instability alters the functions of tumor suppressor genes and oncogenes [1]. MSI has been described in tumors from patients with hereditary nonpolyposis CRC, sporadic CRC, and other types of cancers. MSI is caused by the loss of DNA mismatch repair activity and is detected in about 15% of all CRC; 3% are hereditary, occurring in Lynch syndrome patients, and the other 12% occur in sporadic CRC [2].

This instability results from inactivation of the DNA mismatch repair (MMR) system either by MMR gene mutation or by promoter hypermethylation of the MMR genes. MSI genes in CRC mainly involve mutations in MMR genes like MutL homolog 1 (*MLH1*), MutS homolog 2 (*MSH2*), MutS homolog 6 (*MSH6*), PMS1 homolog 2 (*PMS2*), or epithelial cellular adhesion molecule (*EPCAM*) (encodes a protein that regulates *MSH2*) with mutant forms failing to correct errors within repetitive DNA sequences [3]. Mutant MMR genes are also shown to interrupt the function of other genes [Ginsenoside Rb1 (*GRB1*), Transcription factor 4 (*TCF4*), WNT1-inducible-signaling pathway protein 3 (*WISP3*), Activin receptor type-2A (*ACVR2*), insulin-like growth factor 2 receptor (*IGF2R*), axin inhibition protein 2 (*AXIN2*), and Caudal Homeobox (*CDX*)]. MMR gene mutations lead to mutations in genes regulating cell cycle arrest or apoptosis [caspase 5 (*CASP5*), PR/SET Domain 2 (*PRDM2*), B-cell lymphoma/leukemia 10 (*BCL10*), Phosphatase and tensin homolog (*PTEN*), proliferation-associated protein 2G4 (*PA2G4*), and Fas cell surface death receptor (*FAS*)], as well as DNA repair [methyl-CpG binding domain 4 (*MBD4*), bloom cyndrome (*BLM*), Checkpoint kinase 1 (*CHK1*), mutL homolog 3 (*MLH3*), double strand break repair protein (*RAD50*), MutS Homolog 3 (*MSH3*), and *MSH6*] [3].

Immune response pathways, involving lymphocytes and T cells, were significantly enriched in MSI-H CRC in the TCGA and GEO datasets [all enrichment scores (ES) > 0, *p* < 0.05]. In one study, investigators observed up-regulation of genes related to immune cells (such as B cells, CD4 + T cells, CD8 + T cells, macrophages, neutrophils, and NK cells), genes related to antigen presentation and cytolytic activity [Cluster of differentiation 8A (*CD8A*), Missense mutations in the perforin (*PRF1*), granzyme A (*GZMA*), and Granzyme B (*GZMB*)], other immune related genes [C-X3-C Motif Chemokine Ligand 1 (*CX3CL1*), CXC chemokine ligand 9 (*CXCL9*), and C-X-C motif chemokine 10 (*CXCL10*)], cytokines [Interferon Gamma (*IFNG*), Interleukin-1Beta (*IL1B*), etc.], and tumor necrosis factor receptor superfamily (*TNFRSF*)-related genes. Based on these findings, the investigators predicted MSI-H CRC may have an inflammatory tumor microenvironment and increased sensitivity to immune check-point inhibitors (ICIs) [4].

The association of chronic inflammation with CRC development is well known in ulcerative colitis (UC). However, the role of inflammatory changes in sporadic CRC pathogenesis is less widely appreciated. In a recent study, we showed the association of dysregulation of inflammation-related pathways in sporadic CRC [5]. Previously, we explored the association of differential methylation with MSI in the pathogenesis of CRC [6]. Among the MSS CRC, a paired comparison revealed differentially methylated loci (DML) covering 686 genes at FDR < 0.001 with delta beta ≥ 20%. Similarly, MSI analysis revealed DML involving 2316 genes. An ANOVA model including interactions (Tumor × MSI) revealed 23,322 loci, where the delta beta was different among MSI vs. MSS patients. Our previous study also suggested the potential efficacy of certain ICIs [cytotoxic T-lymphocyte associated protein 4 (*CTLA4*) and Hepatitis A virus cellular receptor 2 (*HAVCR2*) inhibitors] in CRC with MSI [6]. In this study, we compared gene expression in CRC with MSS vs. MSI. We compared differential gene expression between tumors and matched adjacent normal-appearing tissues in patients with MSI CRC vs. MSS CRC. This provides a robust way to control for inter-individual gene expression differences and can accurately detect the genes associated with tumor pathogenesis.

## 2. Materials and Methods

The tissue samples from 71 CRC patients (male = 43 and female = 28) were collected from the Department of Pathology, Bangabandhu Sheikh Mujib Medical University (BSMMU), Dhaka, Bangladesh, from December 2009 to May 2016. These patients were included in our previous studies [5,7,8,9]. Details of sample collection and preservation are described in previous studies [8,9]. For each patient, fresh specimens were collected from resected tumors (referred to as CRC in this article) and surrounding normal-appearing colon tissue (referred to as normal) 5–10 cm from the tumor.

RNA was extracted from tissue preserved in RNA later using the RiboPure kit (Qiagen, Germantown, MD, USA) following manufacturter’s recommended protocol. DNA was extracted from the fresh frozen tissue using the Puregene Core kit (Qiagen, Germantown, MD, USA).

### 2.1. Genome-Wide Gene Expression Assay

We used the Illumina^®^ TotalPrep RNA Amplification Kit (Ambion, a part of Life Technologies Corporation, Carlsbad, CA, USA) for cRNA synthesis. For the transcriptome-wide gene expression assay, we used the Illumina HT12 v4 BeadChip (Illumina Inc., San Diego, CA, USA) that contains a total of 47,231 probes that cover 31,335 genes. A total of 71 paired samples (tumor and adjacent healthy tissue) from CRC patients (m = 43, f = 28) were used. Paired tumor and normal samples were processed on the same chip.

### 2.2. MSI Detection

A high-resolution melting (HRM) analysis method was used for the detection of MSI markers—BAT25, BAT26 and CAT25, as described in earlier studies [8,10]. BAT25 and BAT26 are the most widely used quasi-monomorphic mononucleotide repeats in the Bethesda panel for the identification of MSI [11]. CAT25 was described by Findeisen et al. as displaying a quasi-monomorphic repeat pattern in normal [12]. The efficacy of the CAT25 marker was confirmed by an earlier study [13]. The PCR steps included enzyme activation at 95 °C for 2 min, followed by 5 cycles of denaturation at 95 °C for 15 s, annealing starting at 60 °C for 30 s, extension at 72 °C for 30 s, and an additional 33 cycles of denaturation at 95 °C for 15 s, annealing at 53 °C for 30 s, and extension at 72 °C for 30 s. Before the HRM step, the products were heated to 95 °C for 1 min and cooled to 40 °C for 1 min, to allow heteroduplex formation. HRM was carried out and the data were collected over the range from 60 °C to 95 °C, with a temperature increment of 0.2 °C/s at each 0.05 s [8,10]. A total of 18 tumor samples showed MSI for BAT25 or BAT26 markers, and all were confirmed by CAT25 [6,12,13].

### 2.3. Statistical Analysis

For categorical variables, the chi-square test was used. For continuous variables, a *t*-test or one-way analysis of variance (ANOVA) was used. For expression data, Partek Genomics Suite (version 7.0) (https://www.partek.com/partek-genomics-suite/, accessed on 16 November 2023) was used. In the ANOVA model, the log_2_-transformed gene expression value was used as the response variable (Y), and “Tumor” (tumor or normal), person ID#, and “MSI-status”, were entered as ANOVA factors.

For the paired analysis, we used the following model:

$$Y_{ijk}=\mu +{Tumor}_{i}+{Person}_{j}+\varepsilon_{ijk}$$

where Y_*ijk*_ represents the *k*-th observation on the *i*-th tumor *j*-th individual. *µ* is the common effect for the whole experiment. *ε_ijk_* represents the random error present in the *k*-th observation on the *i*-th tumor *j*-th person. It was assumed that the errors *ε_ijk_* are normally and independently distributed with mean 0 and standard deviation *δ.*

For the detection of the interaction between tumor and MSI status, we used the following model:

$$Y_{ijk}=\mu +{Tumor}_{i}+{Tumor*MSI\;status}_{ij}+\varepsilon_{ijk}$$

where Y_*ijk*_ represents the *k*-th observation on the *i*-th tumor *j*-th MSI status. “μ” is the common effect for the whole experiment. *ε_ijk_* represents the random error present in the *k*-th observation on the *i*-th tumor *j*-th MSI status of CRC.

In Gene ontology (GO) Enrichment analysis, we tested if the genes found to be differentially expressed fell into a GO category more frequently than expected by chance as previously described [14]. The chi-square test was used to compare the “number of significant genes from a given category/total number of significant genes” vs. “number of genes on chip in that category/total number of genes on the microarray chip”.

The gene set ANOVA is a mixed model ANOVA that compares expression levels of a “set of genes” instead of an individual gene in different groups (https://www.partek.com/partek-genomics-suite/, accessed on 16 November 2023). The result is expressed at the level of the “gene set” category by averaging the member genes’ results. The following model was used:

Model: Y = μ + T + P + G + S (T ∗ P) + ε

where Y represents the expression of a “gene set” category, μ is the common effect, T is the tissue-to-tissue (tumor/normal) effect, P is the patient-to-patient effect, G is the gene-to-gene effect (differential expression of genes within that gene set category independent of tissue types), S (T × P) is the sample-to-sample effect, and ε represents the random error. All of the figures were generated using Partek Genomics Suite (version 7.0) (https://www.partek.com/partek-genomics-suite/, accessed on 16 November 2023).

## 3. Results

The characteristics of patients with MSI and MSS CRC are shown in Table 1. For both groups of patients, we used pair-wise comparison of tumor and normal colonic mucosa from the same patient for gene expression profiles.

### 3.1. Gene-Level Analysis

In MSS patients, at the gene-level analysis, 129 genes were differentially expressed (fold change (FC) ≥ 3 at FDR 0.05 level) in tumor tissue compared to adjacent normal tissue. In MSI patients, the number of similarly differentially expressed genes (FC ≥ 3 at FDR 0.05 level) was 190.

**Figure 1:**
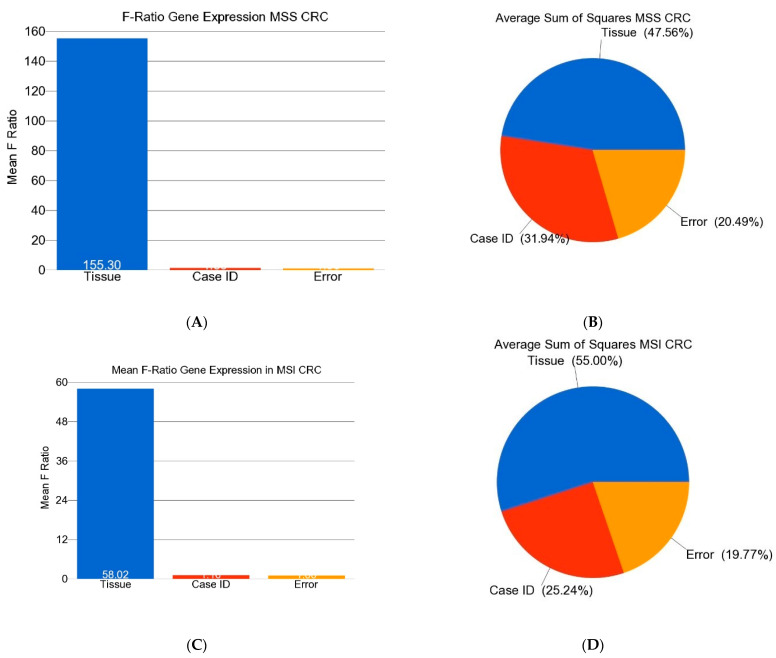
Variation of the differentially expressed genes. Sources of variation in gene expression that can be explained by the ANOVA models are shown. The upper panel (**A**,**B**) shows paired analysis (taking the “Case ID” in the ANOVA model) in MSS patients, and the lower panel (**C**,**D**) shows result of paired analysis for MSI patients. The mean F-ratio (F-statistics for the factor/F-statistics for the model error) shown in the bar graphs (**A**,**C**) represents the significance of the factor in the ANOVA model. The sum of squares in the ANOVA model shown in the pie charts (**B**,**D**) represents the proportion of the variation explained by the indicated factors. It should be noted that in both MSS and MSI patients, more than 25% of the variation in gene expression could be explained by the “Case ID”—indicating interindividual variation.

**Figure 2:**
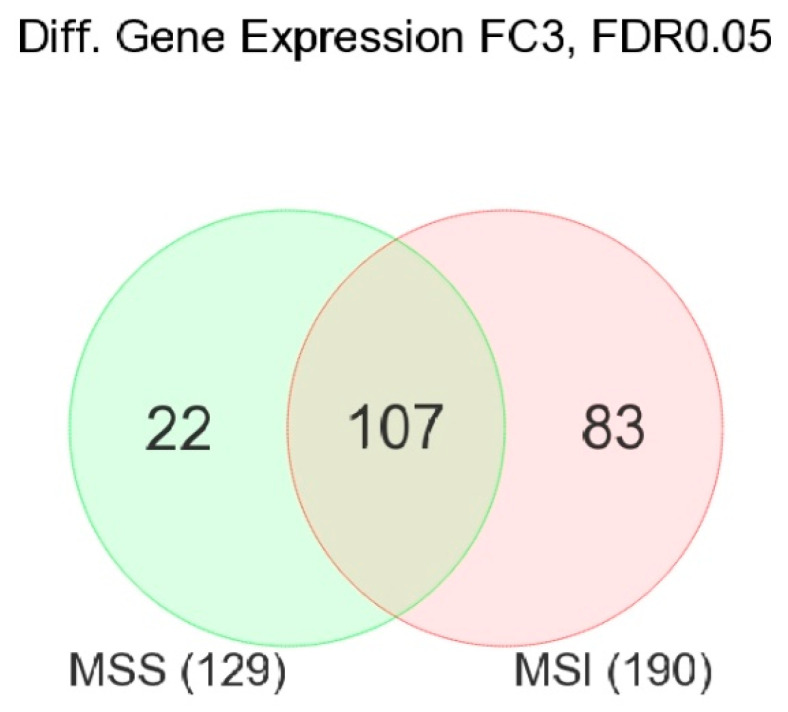
Venn diagram showing the overlap in differentially expressed genes (tumor vs. corresponding normal tissue with FC ≥ 3 at FDR 0.05) in MSS CRC (on left, in light green, n = 129) and in MSI CRC (on right side, in pink, n = 190). Of the 129 differentially expressed genes in MSS group, 107 were also found to be differentially expressed in MSI tumors, and the other 22 were found only in MSS tumors. Of the 190 differentially expressed genes in the MSI group, 107 were common, and the other 83 were found only in MSI tumors.

Figure 1 shows the proportion of the variation explained by interindividual variation—or in other words, the significance of paired observation. It should be noted that in both MSS and MSI CRC patients, inter-individual variation accounted for about one-third to one-fourth of the variation in the gene expression. The overlap of these two gene lists is shown in Figure 2.

A large proportion of the differentially expressed genes (tumor vs. corresponding normal) overlapped in MSS and MSI tumors. There were 107 genes that were differentially expressed in CRC tissue in both MSI and MSS CRC. But, among the differentially expressed genes in MSI tumors (n = 190), 83 genes were differentially expressed only in the MSI group (see Figure 2). In contrast, there were only 22 genes (out of total 129 genes) that were differentially expressed only in MSS tumors.

**Figure 3:**
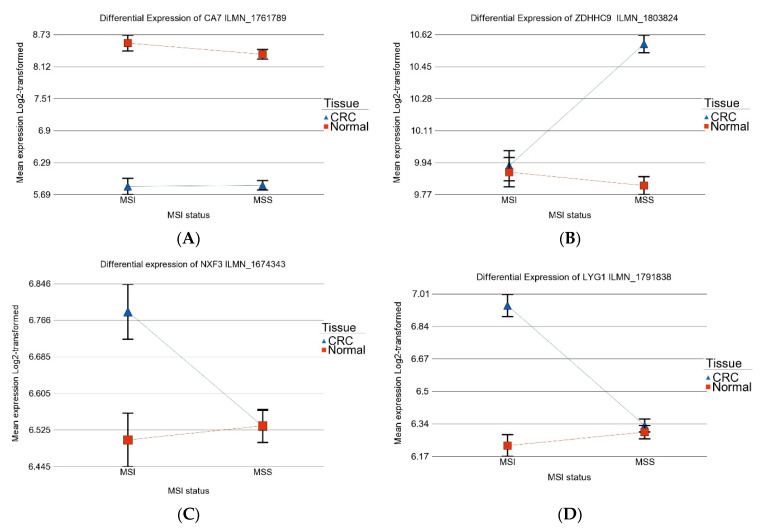
Examples of differentially expressed genes in tumor tissue vs. corresponding normal tissue depending on the MSI status. Upper left panel (**A**) shows the *CA7* gene is down-regulated irrespective of MSI status. Upper right panel (**B**) shows that the gene Zinc Finger DHHC-Type Palmitoyltransferase 9 (*ZDHHC9*) is up-regulated only in MSS tumors; Lower left panel (**C**) and lower right panel (**D**) show up-regulation of nuclear RNA export factor 3 (*NXF3*) and Lysozyme G1 (*LYG1*), respectively, only in MSI but not in MSS CRC.

In other words, some genes were differentially expressed in CRC tissue irrespective of MSI vs. MSS status (the common 107 genes), while other genes were differentially expressed in CRC only in the presence or absence of MSI. Figure 3 shows examples of such genes.

### 3.2. Enrichment Analysis of Differentially Expressed Genes

**Figure 4:**
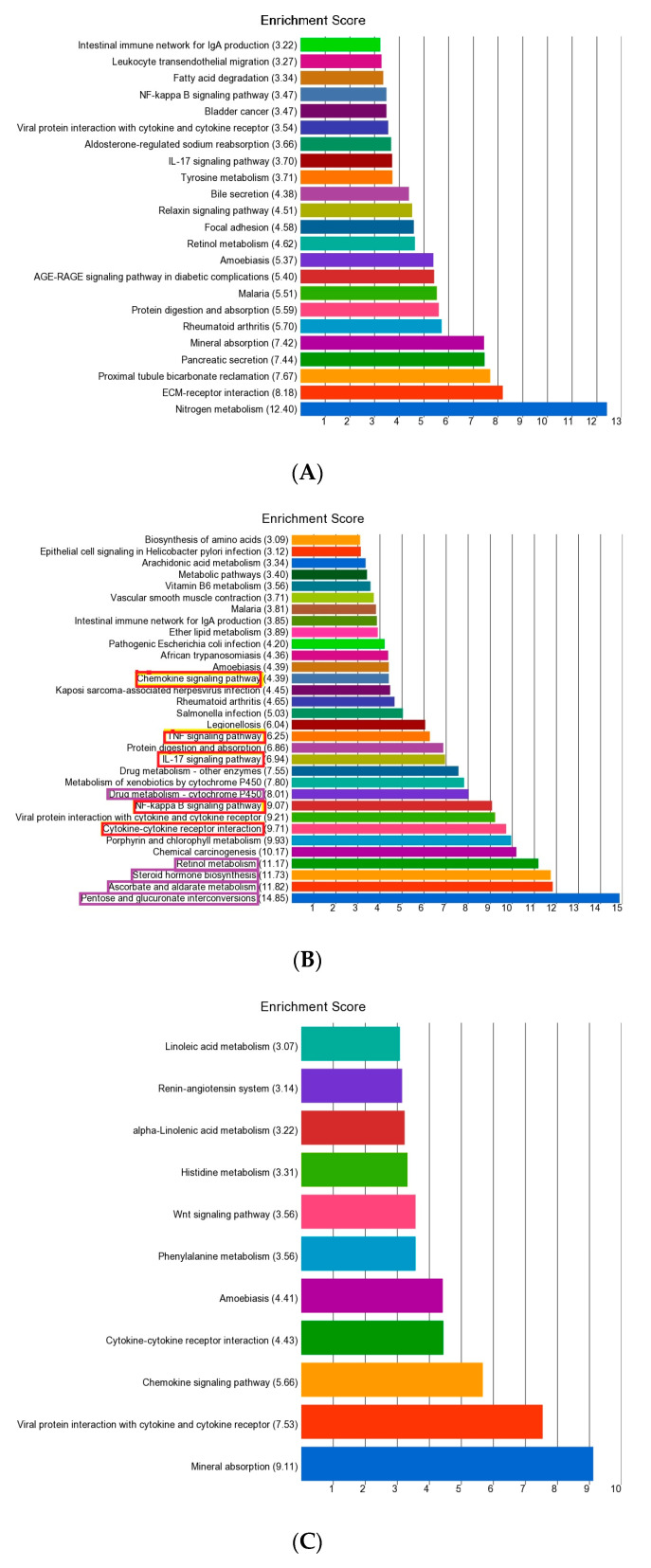
Enrichment analysis of the common 107 genes (shown in Figure 2) is shown in the upper panel (**A**); the enrichment of 83 genes differentially expressed in MSI tumors only is shown in the middle panel (**B**); and the enrichment of 22 genes differentially expressed only in MSS tumors is shown in the lower panel (**C**). The enrichment scores are presented on the *x*-axis, and the pathways are shown on the *y*-axis.

In the next step, we tested whether the list of genes found to be differentially expressed fell into a Gene ontology pathway more often than expected by chance. The result of the enrichment analysis for the “common 107 differentially expressed genes”, the “83 genes differentially expressed only in MSI group” and the “22 genes differentially expressed only in MSS group” are shown in Figure 4A–C, respectively.

We noted there were two major types of pathways enriched for genes differentially expressed only in the presence of MSI: (a) inflammation-related pathways (shown in red in Figure 4B) and (b) metabolism-related pathways (shown in purple in Figure 4B). The genes belonging to the inflammation-related pathways were up-regulated in CRC tissue compared to corresponding normal tissue. On the other hand, genes belonging to the metabolic pathways were down-regulated in the CRC tissue compared to corresponding normal tissue.

In the next step, we examined whether results from gene-level analysis followed by enrichment analysis could be reproduced at the pathway-level analysis.

### 3.3. Gene-Set or Pathway Level Analysis

We used gene-set ANOVA to identify the “set of genes” (we tested the KEGG pathways) that are differentially expressed in the tumor tissue compared to corresponding normal tissue. Basically, we examined if on average, the expression of all the genes in a given pathway were significantly different among tumor-normal pairs. The detailed results of gene-set ANOVA in patients with MSI and MSS CRC are presented in the Supplementary Tables S1 and S2, respectively. Most of the pathways (238 in MSI and 257 in the MSS) were significant at FDR 0.05 level. When we selected the leading 100 from each list of pathways, we found that 77 of the up-regulated pathways were common to both MSI and MSS tumors, indicating shared pathways for both MSI and MSS tumors. In addition to the CRC vs. adjacent normal paired analysis for MSI or MSS tumors separately, by including an interaction term “tissue (tumor or normal) × MSI status (MSI or MSS)” in the gene-set ANOVA model(s), we could include all tumors to test if the magnitude of difference between tumor-normal pairs in MSI tumors was significantly different than the magnitude in MSS tumors. The detailed results of such a model with interaction *p*-values are presented in Supplementary Table S3. The results indicated that compared to normal tissue, some of the top dysregulated pathways were more markedly up-regulated in MSI tumors compared to MSS tumors. Examples include “p53 signaling pathway” [FC 1.08 (95% CI 1.06–1.09) in MSI vs. FC 1.04 (95% CI 1.03–1.05); interaction *p* = 5.91 × 10^−15^] and “TNF signaling pathway” [(FC 1.06 (95% CI 1.05–1.08) in MSI vs. FC = 1.03 (95%CI 1.02–1.03) in MSS; interaction *p* = 1.67 × 10^−6^]. Conversely, some of the top dysregulated pathways were more markedly down-regulated in MSI tumors than in MSS tumors. Examples included “PPAR signaling pathway” [FC = −1.08 (95%CI from −1.11 to −1.07) in MSI vs. FC = −1.05 (95%CI: from −1.06 to −1.04) in MSS; interaction *p* = 0.001], “Peroxisome related genes” [FC = −1.08 (95%CI: from −1.10 to −1.07) in MSI vs. FC = −1.04 (95% CI: from −1.05 to −1.03) in MSS; interaction *p* = 3.45 × 10^−9^], and “fat digestion and absorption pathway” [FC = −1.15 (95%CI: from −1.18 to −1.11) in MSI vs. FC = −1.10 (95% CI: from −1.12 to −1.08) in MSS; interaction *p* = 0.006]. It should be noted that these differences were not detected in gene-level analyses. Genes in “mismatch repair pathway” were up-regulated in both MSI and MSS CRC, but as expected, it was less efficient in MSI patients [FC 1.14 (95% CI 1.11–1.16)] compared to MSS tumors [FC 1.16 (95% CI 1.14–1.17)] with interaction *p* = 5.78 × 10^−7^.

Table 2 shows summary results from such gene-set ANOVA of the same pathways that were found to be enriched in the gene-level analysis shown above. For example, the gene-set ANOVA of all the genes involved in the *IL-17* signaling pathway shows that on average the genes were up-regulated by 1.19 fold (95% CI 1.167–1.21) in patients with MSI CRC and by 1.15 fold (95% CI 1.13–1.16) in patients with MSS CRC, and this magnitude of up-regulation was significantly higher in the presence of MSI (interaction *p* = 0.0009). We could perform gene set ANOVA for 27 of the 29 pathways shown in the enrichment analysis in Figure 4B. We noted that 25 out of 27 pathways showed statistically significant interaction, supporting the fact that the magnitude of differential expression (including the directionality in terms of up- or down-regulation) of these pathways were different in the presence or absence of MSI as we observed in gene-level analysis as well.

**Figure 5:**
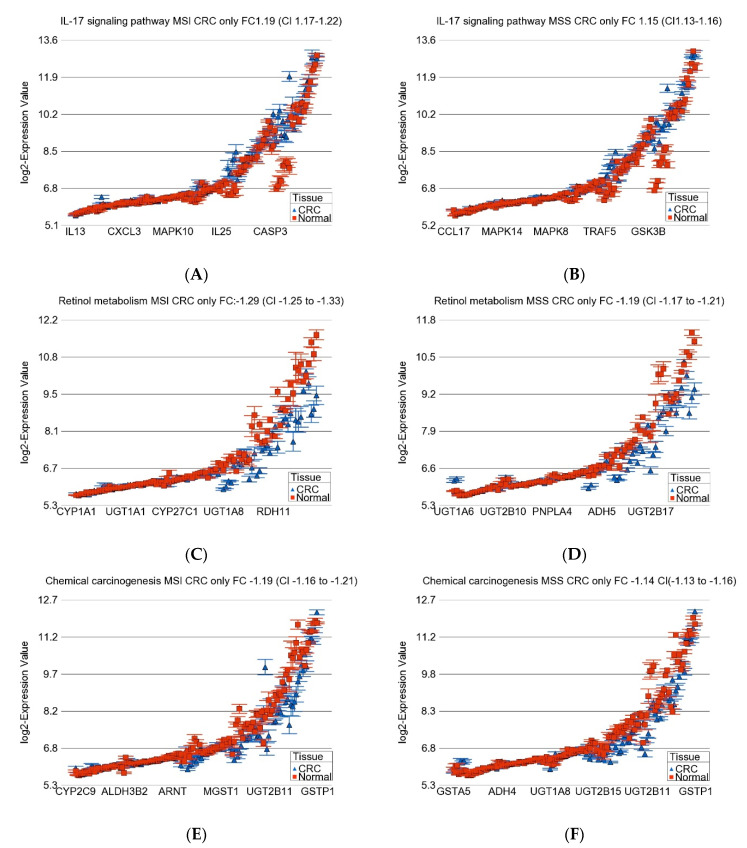
Differential gene expression of genes involved in *IL-17* pathway (upper panel (**A**,**B**)), Retinol metabolism (middle panel (**C**,**D**)), and “chemical carcinogenesis” (lower panel (**E**,**F**)) are shown. Gene expression data for MSI tumors are shown in left panels, and MSS tumors are shown in the right panels. CRC tissue (in blue) is compared to corresponding normal tissue (in red). The *x*-axis represents the gene probes, and the *y*-axis shows their Log_2_ transformed expression value. The average magnitude of overexpression of the *IL-17* pathway was significantly higher (ANOVA interaction *p* = 0.0009) for MSI tumors. The down-regulation of “retinol metabolism pathway” (ANOVA interaction *p* = 0.0001) and genes involved in “chemical carcinogenesis” (ANOVA interaction *p* = 6.15 × 10^−5^) was more marked in MSI tumors.

Figure 5 shows examples of differential expression of some of the top inflammatory and metabolic pathways in MSI and MSS tumors.

In addition to the above-mentioned KEGG pathways, we also asked if the presence or absence of MSI tumor type influenced the different gene sets involved in some known biological processes involved in cancer. The details of these gene-sets are presented in Supplementary Table S4. Genes involved in caspase executor, caspase initiators, and DNA repair showed different magnitudes of differential expression in MSI and MSS tumors. The magnitudes of differential expression in “anti-tumor suppressor genes”, “p-53 suppressors”, “pro-apoptosis genes”, “anti-apoptotic genes”, “growth factor receptor genes” and “tumor suppressor genes” were not statistically different among MSS and MSI tumors.

**Figure 6:**
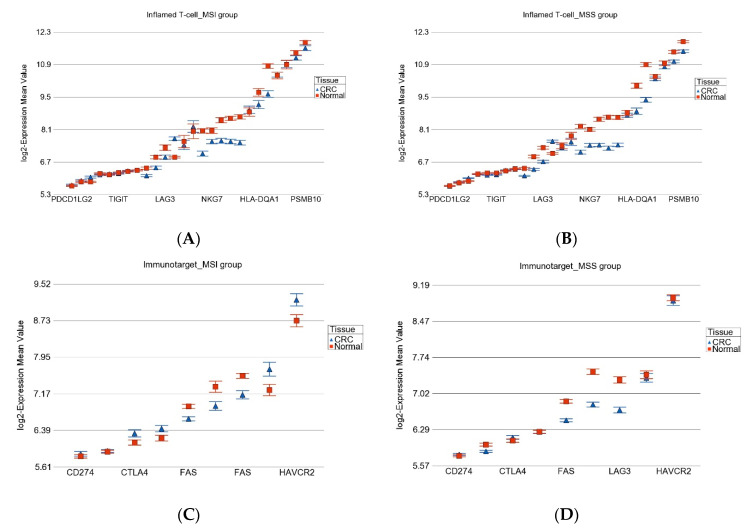
Differential gene expression of T-cell inflamed gene set (upper panel (**A**,**B**)), and some known immune target genes are shown (lower panel (**C**,**D**)). Gene expression data from MSI tumors are presented in the left panels, and those of MSS tumors are shown in the right panels. CRC tissue (in blue) is compared to corresponding normal tissue (in red). The gene probes (multiple probes for some genes) are shown in the *x*-axis and the Log_2_ transformed expression values are shown in *y*-axis.

On average, the inflamed T-cell-related genes were down-regulated by FC = −1.19 (95% CI: −1.24 to −1.13) in MSI tumor tissue compared to FC = −1.31 (95% CI: −1.35 to −1.28) in MSS tumors (interaction *p* = 2.69 × 10^−6^). This more pronounced down-regulation of inflamed T-cell genes in MSS may explain the lack of effectiveness of ICIs in MSS patients (see Figure 6A,B).

Figure 6C,D show that at least in this population, Programmed Cell Death Ligand 1 (*PDL1*) (also known as *CD274*, shown in Figure) was not up-regulated in MSI or MSS tumors, but other immune targets like *CTLA4* and *HAVCR2* were up-regulated in MSI but not in MSS tumor tissue compared to corresponding normal tissue. This suggests there might be a potential beneficial effect of ICIs targeting *CTLA4* and *HAVCR2* but not targeting *CD274* (or *PDL1*).

### 3.4. Differential Gene Expression: MSI vs. MSS Tumors

When we compared gene expression in MSI vs. MSS tumors consistent with several prior studies, a total of 219 probes showed differential expression at FDR 0.05 level. But when we took FC into consideration, only four genes had FC ≥ 2 and all four [Quinolinate phosphoribosyltransferase (*QPRT*), Synaptotagmin 7 (*SYT7*), NADPH quinone oxidoreductase (*NQO1*) and Tumor-associated calcium signal transducer 2 (*TACSTD2*)] were down-regulated in MSI compared to MSS tumors.

### 3.5. Previously Reported Genes in Our Data Set

**Figure 7:**
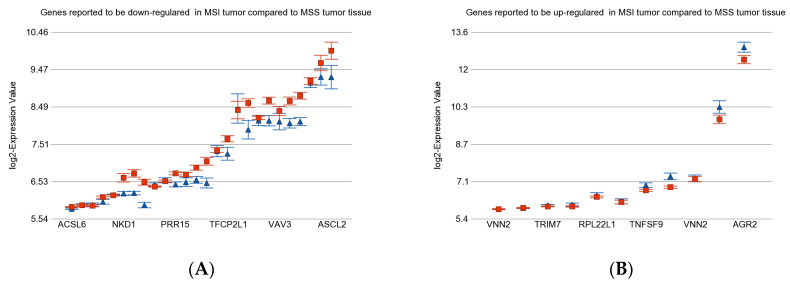
Difference of Gene expression in MSI tumor vs. MSS tumor tissue. The gene list was taken from a previous publication [15] and tested in our data. Gene expression of MSI tumor (in blue) is compared to MSS tumors (in red). Genes reported to be down-regulated in MSI are shown in the left panel (**A**), and genes reported to be up-regulated in previous publication are presented in the right panel (**B**).

We also checked the expression patterns of previously published gene sets against our dataset. The gene signature was used to differentiate MSI tumor tissue from MSS tumor tissue [15]. The details are presented in Supplementary Table S5. Most genes reported to be down-regulated in MSI compared to MSS tumors in a previous publication [15] were also found to be down-regulated in our data set (see Figure 7A). A few of the genes reported to be up-regulated in MSI tumors compared to MSS tumors in a previous publication were also found to be up-regulated in our data set (see Figure 7B).

**Figure 8:**
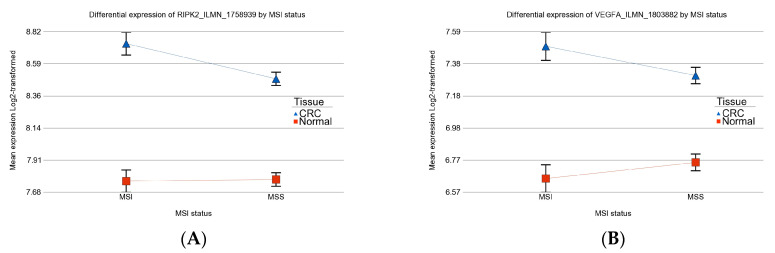
Differential expression of *RIPK2* (on the left, (**A**)) and *VEGFA* (on the right, (**B**)) in CRC tissue (in blue) compared to normal tissue (in red) by MSI status.

In a recent paper, RF Jaafar et.al reported that a high expression of receptor-interacting protein kinase 2 (*RIPK2*) was associated with a high expression of vascular endothelial growth factor (*VEGFA*) and increased mortality in CRC [16]. *RIPK2* has a critical role in immune and inflammatory pathways. In our data set, both of these genes were significantly up-regulated in CRC tissue compared to normal tissue (see Figure 8). Interestingly, the magnitude of up-regulation of *RIPK2* was greater (interaction *p* = 0.03) in MSI tumors [FC 1.97 (CI 1.69–2.3)] compared to MSS tumors [FC 1.64 (CI 1.5–1.8)]. In contrast, the magnitudes of up-regulation in *VEGFA* in MSI tumors [FC1.79 (CI 1.53–2.13)] and in MSS tumors [FC 1.1.47 (CI 1.33–1.63)] were not different (interaction *p* = 0.13).

## 4. Discussion

In CRC pathogenesis, MSI is a known oncogenetic marker. This is more frequently encountered in right-side CRC and has implications for both prognosis and therapy. Several prior studies reported gene expression profiles comparing MSI and MSS cell lines and/or tumor tissue [15,17,18,19]. To our knowledge, however, this is the first study to compare tumor and adjacent normal-appearing colonic tissue from same individual in a relatively homogenous Bangladeshi population. Here we have explored the association of differential gene expression and MSI status in the pathogenesis of CRC. Individual gene-level and pathway-level analyses of our study indicate that a large proportion of the dysregulated genes or pathways are common to both MSI and MSS tumors. We also found, however, that a substantial proportion of dysregulated genes or pathways are more markedly altered in MSI tumors compared to MSS tumors (each normalized to their matched adjacent tissue).

Our findings from pathway enrichment analysis, which takes lists of differentially and significantly expressed genes from gene-level analysis into account, suggested the possible associations of (a) up-regulated inflammation-related pathways (*TNF* signaling pathway, NF-kappa B signaling pathway, *IL-17* signaling) and (b) down-regulated metabolism-related pathways (retinol metabolism, steroid hormone biosynthesis, and ascorbate and aldarate metabolism) in MSI compared to MSS tumors. These findings were consistent with the pathway-level analysis using gene-set ANOVA where we compared the average expression of all the genes included in a given pathway. These dysregulated pathways are suggested as future targets for therapeutic interventions. Down-regulation of fatty acid metabolism genes in CRC have been shown in a previous study [20].

One of our reviewers raised an important issue regarding the difference in *TP53* mutation status among the MSI and MSS tumors. Unfortunately, the *TP53* mutation status was not available. Depending on funding, we will try to do this in future. However, we looked at the expression of *TP53* in the data set. In both groups, there was significant down-regulation of *TP53* in CRC tissue compared to normal tissue. In the MSI group, the FC = −1.17 (95% CI: −1.05 to −1.29, *p* = 0.003), and in MSS group, the FC = −1.22 (95% CI: −1.15 to −1.30, *p* = 2.56 × 10^−9^). The magnitude of down-regulation was more marked in the MSS compared to MSI (interaction *p* = 0.01).

The therapeutic landscapes in CRC were reviewed in a recent paper [21]. Prior studies suggest the limited efficacy of ICIs targeting PD-1 in patients with MSS CRC [22,23]. *IL-17A* increases *PD-L1* protein expression and may promote resistance to anti-PD-1 therapy. In the murine model of CRC, it was shown that blocking *IL-17A* could improve the efficacy of anti-PD-1 therapy in MSS CRC [22]. In our study, we observed an increase in IL17 but did not see an increase in PDL1 (*CD274*) mRNA expression in CRC tissue. A previous cell-line based study also found that IL-17 did not induce PDL1 mRNA expression but up-regulated PDL1 protein expression in HCT116 and LNCaP cells [24]. Data from the current study may also help explain why there is an unsatisfactory response to anti-PD1 therapy in CRC, and it also sheds light on new pathways to target in MSS tumors.

There are other important implications of MSI in CRC therapies. In this regard, the literature has mixed views on the prognostic value of the MSI phenotype in predicting the response rate to irinotecan regimens in metastatic CRC patients. A few experimental and retrospective studies have reported higher sensitivity of the MSI CRC phenotype to irinotecan therapy [25,26,27].

A study by Jacob et al. showed that CRC cell lines defective in DNA MMR exhibit increased sensitivity to camptothecin and etoposide [28]. Cytokeratin 20 (*CK20*) and mitogen-activated protein kinase kinase kinase 8 (*MAP3K8*) up-regulations were observed in MSS tumors and associated with lymph node metastasis, recurrence, and/or distant metastasis and short median survival [29]. In CRC, interleukin-25 (*IL-25*) and interleukin-33 (*IL-33*) are predominantly expressed by transformed colonocytes compared to the adjacent normal colonocytes [30]. These cytokines shape the tumor microenvironment and can promote or inhibit CRC development depending on the specific CRC subtype [31,32].

High expression of cleaved caspase-3 (*CC3*) in CRC may be associated with a good prognosis [33]. This may be because of the apoptosis in the tumor-associated stroma. [33].

Investigators screened different molecular types of CRC on cell lines to find the ones with the highest sensitivity to curcumin, a natural product [34]. Most curcumin-sensitive cell lines were of the MSS molecular type, and they had high baseline levels of the IκBα protein. Conversely, curcumin-resistant cell lines were mainly of the MSI type, and showed low baseline IκBα levels [34]. This suggests curcumin could be useful for treatment or prevention of MSS CRC, and there are ongoing efforts to increase its bioavailability [35].

In a large study, gene expression data from 63 MSI-H CRC were compared to 550 MSS CRC [36]. Compared to MSS, in the MSI tumor, there were 409 significantly differentially expressed genes—302 were down-regulated and 107 were up-regulated without taking the fold-change into consideration. Multiple known oncogenes like insulin-like growth factor 2 (*IGF2*), fibroblast growth factor 3 (*FGF3*), and claudin 18 (*CLDN18*) were down-regulated [36]. But when the fold change in expression was considered, out of the 107 up-regulated genes, only one gene, regenerating family member 3 gamma (*REG3G*), had a log_2_FC > 2. So, the number of significant differentially expressed genes in a given gene list depends on the criteria of selection. Consistent with this small number of genes, comparison of MSI and MSS tumors in our data set yielded only 4 genes with FC > 2 at FDR 0.05.

In the same study by Xu et al. [36], among the 63 MSI-H samples, 21 were metastatic and the remaining 42 were non-metastatic. When these two groups were compared, it was found that in the metastatic MSI-H CRC group, 14 genes were up-regulated, and 20 genes were down-regulated compared to the non-metastatic group. The down-regulated genes included palmitoleoyl-protein carboxylesterase (NOTUM), which is known to affect the Wnt signaling pathway, and serpin family B member 2 (*SERPINB2*), a gene related to autophagy and senescence in cancer. The up-regulated genes included serum amyloid A1 (*SAA1*), a known tumor biomarker [36].

We also examined our data set for several genes previously reported to be differentially expressed in MSI CRC compared to MSS CRC [15]. When we compared the MSI and MSS tumors in our data set, our results were mostly consistent with the previous findings [15].

We acknowledge several limitations of the present study. First, we did not have any CRC patients with distant metastasis. Second, we also do not have somatic mutation data for the MMR genes, which might have allowed us to see associations of specific MMR mutations with gene expression changes. Third, we also do not have any clinical follow-up on responses to chemotherapy or immunotherapy. However, we have looked for a molecular basis in tumors for the potential efficacy of ICIs in these MSI and MSS CRC patients.

Acknowledging these limitations, we wish also to emphasize several strengths of our study. First, we used paired tumor and normal samples from the same individual for comparison, which is the most robust method for detecting any differential gene expression changes in cancer pathogenesis as it controls for variance in normal tissues among different individuals. Second, we used tissue samples preserved in RNA later for RNA and fresh frozen samples for DNA, which are the gold standards for preserving RNA and DNA for down-stream analysis. Third, to our knowledge, this is one of the first studies involving Bangladeshi subjects with CRC to comprehensively assess differences between MSI and MSS tumors in CRC pathogenesis from the molecular perspective. Detection of MSI in the CRC tissue reflects a structural alteration that derives from defective DNA repair mechanisms involved in cancer. Our studies further expand the understanding of DNA mismatch repair defects and dysregulation of gene expression pathways in CRC.

## 5. Conclusions

We document the association between MSI status and differential gene expression in CRC compared to normal tissue, which helps to broaden our understanding of CRC pathogenesis. Furthermore, targeting one or more of these dysregulated pathways could provide the basis for personalized improved therapies for MSI and MSS CRC.

## Figures and Tables

**Table 1:** Clinical and pathological characteristics of the 71 CRC patients.

Characteristic	Category	MSI	MSS	Chi Square *p*-Value
Sex	Male	4	24	0.14
	Female	14	29	
Location	Distal	11	47	0.023
	Proximal	7	6	
Stage	Stage-1	3	9	0.68
	Stage-2	7	15	
	Stage-3	8	29	
Tumor Grade	Low	14	43	1
	High	4	10	
LymphNode	Present	8	29	0.63
	Absent	10	24	
TIL	0	12	27	0.37
	1	6	26	
Signet ring	Absent	14	37	0.72
	Present	4	16	
LVinvasion	Absent	12	24	0.19
	Present	6	29	
PNinvasion	Absent	15	40	0.72
	Present	3	13	
KRAS	Mutant	5	15	0.79
	Wild	13	38	
BRAF	Mutant	1	0	0.56
	Wild	17	53	

Abbreviations: TIL tumor infiltrating lymphocytes; LV lymphovascular; PN perineural.

**Table 2:** Gene-set ANOVA for the pathways found in the enrichment analysis.

			MSI			MSS	
Gene Sets	Interaction	Fold Change	(95% CI)	*p*	Fold Change	(95% CI)	*p*
	*p*						
IL-17 signaling pathway	0.000957916	1.19	(1.16–1.21)	1.02 × 10^−59^	1.15	(1.13–1.16)	8.11 × 10^−104^
Retinol metabolism	0.000177561	−1.27	(−1.31–1.23)	7.35 × 10^−56^	−1.20	(−1.21–1.17)	1.19368 × 10^−86^
Chemical carcinogenesis	6.15 × 10^−5^	−1.18	(−1.21–1.15)	1.11 × 10^−48^	−1.15	(−1.16–1.13)	1.66064 × 10^−90^
Drug metabolism—cytochrome P450	0.000231622	−1.22	(−1.25–1.18)	5.92 × 10^−43^	−1.17	(−1.19–1.15)	1.74586 × 10^−76^
Intestinal immune network for IgA production	2.37 × 10^−8^	−1.15	(−1.18–1.10)	9.73 × 10^−16^	−1.29	(−1.31–1.26)	7.7175 × 10^−132^
Steroid hormone biosynthesis	0.000179774	−1.22	(−1.25–1.18)	1.01 × 10^−41^	−1.14	(−1.16–1.12)	6.24018 × 10^−53^
Pentose and glucuronate interconversions	3.37 × 10^−6^	−1.35	(−1.40–1.28)	2.24 × 10^−40^	−1.23	(−1.26–1.19)	1.17089 × 10^−54^
Metabolism of xenobiotics by cytochrome P450	0.000177715	−1.17	(−1.20–1.14)	3.48 × 10^−32^	−1.14	(−1.15–1.11)	1.60731 × 10^−56^
Vascular smooth muscle contraction	2.30 × 10^−6^	−1.07	(−1.08–1.05)	2.87 × 10^−27^	−1.06	(−1.06–1.05)	3.17997 × 10^−54^
Ether lipid metabolism	0.591294	−1.13	(−1.15–1.10)	6.57 × 10^−23^	−1.11	(−1.12–1.09)	1.49968 × 10^−47^
Ascorbate and aldarate metabolism	8.54 × 10^−7^	−1.29	(−1.35–1.23)	1.31 × 10^−26^	−1.20	(−1.22–1.16)	1.9392 × 10^−35^
Porphyrin and chlorophyll metabolism	4.22 × 10^−5^	−1.17	(−1.20–1.13)	3.23 × 10^−22^	−1.12	(−1.14–1.10)	2.08756 × 10^−33^
Salmonella infection	3.63 × 10^−7^	1.11	(1.08–1.12)	8.10 × 10^−25^	1.06	(1.04–1.07)	1.21337 × 10^−24^
Protein digestion and absorption	0.0136228	1.08	(1.05–1.09)	1.34 × 10^−15^	1.08	(1.06–1.09)	9.45574 × 10^−42^
Amoebiasis	0.0187527	1.09	(1.07–1.11)	4.57 × 10^−19^	1.06	(1.04–1.06)	1.29366 × 10^−21^
Drug metabolism—other enzymes	2.02 × 10^−8^	−1.11	(−1.13–1.08)	3.14 × 10^−18^	−1.06	(−1.07–1.04)	3.68932 × 10^−19^
Legionellosis	8.09 × 10^−8^	1.11	(1.07–1.13)	9.76 × 10^−17^	1.06	(1.04–1.07)	1.91509 × 10^−16^
Malaria	0.000186005	1.18	(1.13–1.21)	1.12 × 10^−18^	1.08	(1.05–1.10)	8.28788 × 10^−12^
Biosynthesis of amino acids	3.22 × 10^−12^	1.06	(1.03–1.07)	2.82 × 10^−10^	1.05	(1.04–1.06)	1.95178 × 10^−24^
TNF signaling pathway	1.67 × 10^−6^	1.06	(1.04–1.07)	3.71 × 10^−18^	1.03	(1.01–1.03)	2.34661 × 10^−9^
Rheumatoid arthritis	4.08 × 10^−17^	1.15	(1.12–1.17)	2.39 × 10^−26^	1.02	(1.00–1.03)	0.0193784
Epithelial cell signaling in Helicobacter pylori infection	0.52238	1.05	(1.03–1.07)	1.18 × 10^−8^	1.04	(1.03–1.05)	1.09674 × 10^−13^
Vitamin B6 metabolism	0.00322795	1.14	(1.05–1.21)	0.000387	1.12	(1.07–1.16)	7.67061 × 10^−8^
Arachidonic acid metabolism	0.059206	−1.02	(−1.04–1.00)	0.0502923	−1.02	(−1.03–1.01)	0.000402162
Viral protein interaction with cytokine and cytokine receptor	2.43 × 10^−9^	1.02	(−1.00–1.03)	0.120965	−1.06	(−1.06–1.04)	6.93335 × 10^−18^
NF-kappa B signaling pathway	9.57 × 10^−7^	1.02	(1.00–1.03)	0.0397081	−1.03	(−1.04–1.02)	1.01891 × 10^−11^
African trypanosomiasis	0.0268193	1.04	(1.00–1.07)	0.0214121	−1.01	(−1.03–1.00)	0.171386

## Data Availability

All supporting data are presented in the tables presented in the main manuscript and as Supplemental Materials.

## Supplementary Materials

The following supporting information can be downloaded at: https://www.mdpi.com/article/10.3390/medicina60030348/s1; Table S1: Gene set ANOVA using KEGG pathways for MSI patients; Table S2: Gene set ANOVA using KEGG pathways for MSS patients; Table S3: Gene set ANOVA using KEGG pathways with interaction term “Tissue × MSI” status; Table S4: Gene set ANOVA using cancer related gene set for all patients; Table S5: Gene expression of the previously reported genes in our data set.
